# TRAJECTORIES OF INTRINSIC CAPACITY AND IMPACT ON ADVERSE OUTCOMES IN COMMUNITY OLDER ADULTS: A LONGITUDINAL STUDY

**DOI:** 10.1093/geroni/igad104.2783

**Published:** 2023-12-21

## Abstract

**Background:**

Maintaining and delaying Intrinsic capacity(IC) in older adults is key to healthy ageing. Determining the point at which IC occurs and declines to delay its progression and intervene in a timely manner is critical. This study aims to explore the trajectories and the critical point of the trajectory changes among the elderly in the Chinese community.

**Methods:**

The data used in this study are from the ongoing national longitudinal study CHARLS, which surveys the elderly population in China. A total of 3901 older adults from the cohort study were included. Logistic regression, group-based trajectory models (GBTM) were used to identify potential heterogeneity in longitudinal changes over time and investigate associations between baseline predictors and trajectories for different cohort members. Results Four different trajectories of IC in older people, namely, Increase (15.7%), Stable (52.7%), and Decline (31.6%) were found. We found several predictors that predict the trends in IC. Age, gender, living area, BMI, self-reported health states, the number of chronic diseases and initial IC scores significantly impact the IC change rate over time. Decreased cognitive function, psychology status and locomotion at baseline predicted new ADL and IADL dependence after 5 years.

**Conclusion:**

The nature of genetics and environmental factors were associated with different trajectory groups of intrinsic capacity, which could help to guide the timing and focus of prevention strategies. Early prevention or intervention of the determinants of these trajectories can maintain or delay the rate of decline in intrinsic capacity and improve healthy ageing.

